# The complete chloroplast genome of *Rhododendron datiandingense* (Ericaceae)

**DOI:** 10.1080/23802359.2021.1931504

**Published:** 2021-05-24

**Authors:** Zheng-Feng Wang, Hui-Fang Feng, You-Yu Li, Hui-Feng Wang, Hong-Lin Cao

**Affiliations:** a Key Laboratory of Vegetation Restoration and Management of Degraded Ecosystems, South China Botanical Garden, Chinese Academy of Sciences, Guangzhou, China; b Center for Plant Ecology, Core Botanical Gardens, Chinese Academy of Sciences, Guangzhou, China; c Southern Marine Science and Engineering Guangdong Laboratory (Guangzhou), Guangzhou, China; d Forest Resources Conservation Center of Guangdong Province, Guangzhou, China; e Guangdong Yunkaishan National Nature Reserve, Maoming, China; f Guangzhou Linfang Ecology Co., Ltd., Guangzhou, China

**Keywords:** *Rhododendron datiandingense*, chloroplast, genome assembly, next generation sequencing

## Abstract

*Rhododendron datiandingense* is newly reported and endemic to China. The genome of *R. datiandingense* is 207,311 bp in length, including a large single-copy region of 190,689 bp and a small single-copy region of 2582 bp, a pair of inverted repeat regions (IRA) of 7020 bp each. The genome encodes 110 genes, comprising 77 protein-coding genes, four ribosomal RNA genes, and 29 transfer RNA genes. Repeat analysis revealed 62 simple sequence repeats (SSRs) in the genome. Phylogenetic analysis revealed that *R. datiandingense* is clearly separated from the other *Rhododendron* species and shown in the basal position.

*Rhododendron* is the largest and extremely diverse genus in the heath family (Ericaceae) (Dai et al. 2020). It includes about 500 species in China, and more than 400 of them are endemic to China (Yang et al. 2020). It is naturally distributed in the North Temperate Zone, preferring moist acid soil, and northeastern Asia is its ancestral area (Yang et al. 2020). *Rhododendron* species are notable for their attractive flowers and handsome foliage and is available for garden use, but it is also used in beverages, such as azalea and Labrador tea. *Rhododendron datiandingense* Z. J. Feng is a newly reported species (Feng et al. 1996). Unlike some widely distributed congeneric species, *R. datiandingense* has a restricted distribution in southern China and is only found in the Guangdong Yunkaishan National Natural Reserve. Field investigation indicated that less than a hundred of individuals were found. It is considered a vulnerable species, and therefore, we report its complete chloroplast genome to provide a germplasm resource for better future conservation.

Fresh leaves of *R. datiandingense* were collected from the Guangdong Yunkaishan National Natural Reserve, Maoming City, China (22°17′50″N, 111°12′30″E). A voucher specimen was deposited at the Herbarium of South China Botanical Garden (Fei-Yan Zeng, zengfeiy@scib.ac.cn) with no. IBSC 0858371. The genomic DNA of *R. datiandingense* was extracted by the CTAB (cetyltrimethylammonium bromide) method, and the extracted DNA was used to construct one short-read sequencing libraries. The libraries were sequenced using the Illumina HiSeq X Ten system. The chloroplast genome of *R. datiandingense* was assembled by Fast-Plast 1.2.8 (McKain and Wilson 2017). The assembled genome was then polished by Pilon 1.24 (Walker et al. 2014) and annotated with CPGAVAS2 (Shi et al. 2019), GeSeq (Tillich et al. 2017), and PGA (Qu et al. 2019). The genome and its annotated genes were submitted to GenBank under the accession number MW381788. Phylogenetic analysis for *R. datiandingense* and the other 16 species was carried out with PhyloSuite 1.2.2 (Zhang et al. 2020) using the concatenated protein sequences of 77 chloroplast coding genes in their chloroplast genomes. PhyloSuite is an integrated phylogenetic analysis platform. Our performance, which used it, included sequence alignment with MAFFT 7.313 (Katoh et al. 2002), alignment trimming with trimAl 1.2 (Capella-Gutiérrez et al. 2009), model selection with ModelFinder (Kalyaanamoorthy et al. 2017), and maximum-likelihood phylogenetic inference with MrBayes 3.2.6 (Ronquist et al. 2012) using *Nymphaea thermarum* and *Oryza sativa* as outgroup species (Liu, Fu, et al. 2020).

The assembled chloroplast genome of *R. datiandingense* was 207,311 bp in length. Its GC content was 36.06%. The genome consisted of a large single-copy region of 190,689 bp and a small single-copy region of 2582 bp, two inverted repeat regions (IRA) with 7020 bp each. The chloroplast genome encoded a total of 110 genes, including 77 protein-coding genes, four ribosomal RNA genes, and 29 transfer RNA genes. A total of 62 simple sequence repeats (SSRs) were identified in the *R. datiandingense* chloroplast genome (Table S1). Among them, the number of mononucleotide-repeat SSRs was 57, dinucleotide-repeat SSRs was 3, and trinucleotide-repeat SSRs was 2. Phylogenetic analysis revealed that genus *Rhododendron* was sister to genus *Vaccinium* and they were both highly supported as monophyletic, which agreed with previous phylogenetic studies (Shen et al. 2019; Liu, Fu, et al. 2020; Liu, Chen, et al. 2020; Ma et al. 2021). Within the genus *Rhododendron*, *R. datiandingense* was clearly separated from the other six *Rhododendron* species and shown in the basal position (Figure 1).

**Figure 1.**
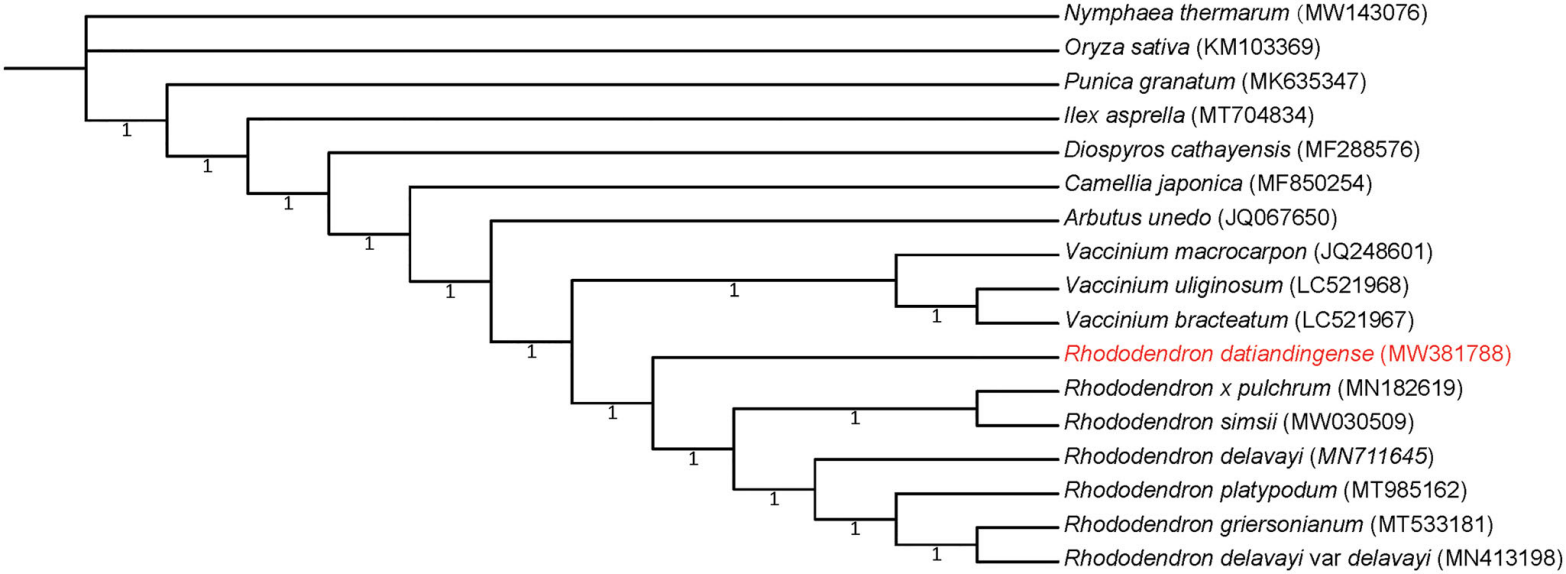
Phylogenetic relationship for *Rhododendron datiandingense* and 16 additional species using their complete chloroplast genomes. The genomes of additional species were downloaded from GenBank and their GenBank accession numbers are shown in parentheses. The numbers on the branches are Bayesian posterior probabilities.

## Data Availability

The complete chloroplast genome sequences of *Rhododendron datiandingense* have been deposited in GenBank under the accession number MW381788 and is also accessible at https://doi.org/10.13140/RG.2.2.25447.78243. The associated BioProject, SRA, and Bio-Sample numbers for short reads are PRJNA686449, SRR13808853, and SAMN17118256.
